# An epidemic model for non-first-order transmission kinetics

**DOI:** 10.1371/journal.pone.0247512

**Published:** 2021-03-11

**Authors:** Eun-Young Mun, Feng Geng

**Affiliations:** 1 Department of Health Behavior and Health Systems, School of Public Health, University of North Texas Health Science Center, Fort Worth, TX, United States of America; 2 School of Professional Studies, Northwestern University, Chicago, IL, United States of America; Universidad Rey Juan Carlos, SPAIN

## Abstract

Compartmental models in epidemiology characterize the spread of an infectious disease by formulating ordinary differential equations to quantify the rate of disease progression through subpopulations defined by the Susceptible-Infectious-Removed (SIR) scheme. The classic rate law central to the SIR compartmental models assumes that the rate of transmission is first order regarding the infectious agent. The current study demonstrates that this assumption does not always hold and provides a theoretical rationale for a more general rate law, inspired by mixed-order chemical reaction kinetics, leading to a modified mathematical model for non-first-order kinetics. Using observed data from 127 countries during the initial phase of the COVID-19 pandemic, we demonstrated that the modified epidemic model is more realistic than the classic, first-order-kinetics based model. We discuss two coefficients associated with the modified epidemic model: transmission rate constant *k* and transmission reaction order *n*. While *k* finds utility in evaluating the effectiveness of control measures due to its responsiveness to external factors, *n* is more closely related to the intrinsic properties of the epidemic agent, including reproductive ability. The rate law for the modified compartmental SIR model is generally applicable to mixed-kinetics disease transmission with heterogeneous transmission mechanisms. By analyzing early-stage epidemic data, this modified epidemic model may be instrumental in providing timely insight into a new epidemic and developing control measures at the beginning of an outbreak.

## Introduction

In epidemic control, speedy action guided by the knowledge of pathogens is crucial. Mathematical models have become essential tools for understanding infectious diseases since the early 20^th^ century [1]. A critical challenge in modeling epidemics is how to gain insight into the intrinsic mechanism of disease transmission during the early stages of epidemics when there are limited data [2,3].

**Fig 1:**
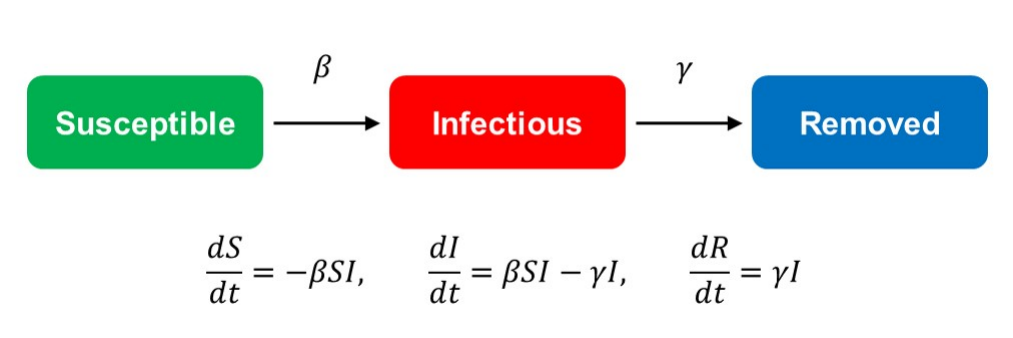
The SIR scheme and associated ordinary differential equations.

The Susceptible-Infectious-Removed (SIR) model is based on a scheme that compartmentalizes the population into susceptible (S), infectious (I), and removed (R) subpopulations [4]. Coefficients and ordinary differential equations are used to quantify the transformation of subjects from one subpopulation to another (Fig 1). Generally, coefficients in these equations are solved numerically or analytically, and the course of epidemics can be predicted via simulation.

Model coefficients, such as β and γ, illustrate the properties of infectious diseases by quantifying the progression rates. In addition, epidemiological indices, such as the basic reproduction number *R*_*0*_, can be derived from these coefficients [1,5,6]. *R*_*0*_ is defined as the average number of secondary cases produced by one infectious agent during the whole infectious period in a fully susceptible population. It quantifies the transmission potential of an infectious disease and is easy to understand conceptually. As such, *R*_*0*_ provides a point of reference for other epidemics and helps detect heterogeneous conditions and populations, in which *R*_*0*_ may take on different values [7].

Environmental factors (e.g., contact structure heterogeneity) and intervention measures (e.g., social distancing and contact tracing) introduce complexity to the natural course of an epidemic, which makes it challenging to estimate *R*_*0*_ [8,9]. Therefore, the effective reproduction rate *Re* has often been used instead. *Re* is a dynamic index of real-time disease status, and when used with *R*_*0*_, it can provide a comparative reference [9,10].

**Fig 2:**
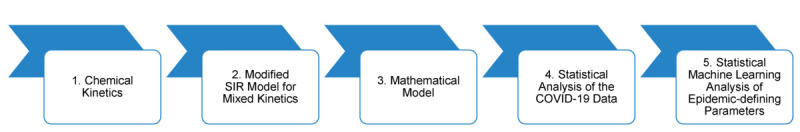
An overview of the current study.

The current study proposes a modified mathematical model based on the modified SIR scheme. Like many previous studies, the mathematical model proposed in the current study derives inspiration from chemical reaction kinetics [11–13], with a critical difference in that the transmission "reaction" is not assumed to be first-order regarding the infectious population. The modified model provides two disease-describing parameters: transmission rate constant *k* and transmission rate order *n*. *k* responds to external intervention measures, such as disease control measures, whereas *n* is conceptually linked to the intrinsic properties of the epidemic agent, such as the reproduction number. Fig 2 provides an overview of the current study.

### The SIR model

The differential equations in Fig 1 depict a disease progression dynamic parallel to an autocatalytic chemical reaction, with the subpopulations *S*, *I*, and *R* representing different reactive molecular species in the reaction mixture. The disease spreading process can be treated as a reaction converting the “reactant” *S* into *I*, where the infectious agent *I* is both the product and the catalyst.

Based on reaction schemes shown in Eqs (1) and (2), the classic rate law for infectious case number change is expressed in Eq (3):

$$S+I\overset{k}{\to }I+I$$ (1)

$$I\overset{k_{r}}{\to }R$$ (2)

$$\frac{d[I]}{dt}=k[S][I]-k_{r}[I]$$ (3)

where [*I*] and [*S*] are the population density of the infected (or infectious) and susceptible individuals, respectively, while *k* and *k*_*r*_ are the reaction rate constants, respectively, for infection and removal.

In chemical reaction terms, Eq (3) describes a first-order reaction kinetics in the infectious agent *I*, *and*, *in the early stages of the epidemics*, *leads to* an exponential growth of [*I*] over time *t*. However, it has been noted that this exponential growth trajectory does not fit real-world data well [14–17]. Various statistical strategies have been used to address this discrepancy, including the adoption of a time-dependent rate constant *k* [10,17].

The current paper draws from a more general framework pioneered by Wilson and Worcester [18], where a transmission rate is not linearly proportional to *S* and *I* (i.e., exponential growth) but instead follows a more generally applicable rate raw. In other words, the rate of transmission is not β[*S*][*I*] but β[*S*]^*p*^[*I*]^*q*^ [19,20], where *p* and *q* are positive constants. It has been discussed why infectious disease outbreaks do not grow exponentially [20,21]. Some later studies have adopted this approach and incorporated *p* and *q* as “decelerating” parameters to improve model fit [22,23]. In the current study, we provide a theoretical rationale for the modified rate law by drawing from chemical kinetics and demonstrate the modified model by analyzing observed data from 127 countries during the initial phase of the COVID-19 pandemic.

### A modified mathematical model with non-first-order transmission kinetics

In a reaction rate equation, the power to which the concentration of a species is raised is called the order of the reaction with respect to that species [24]. Reaction order is an empirical value deduced from observed data, and reaction mechanism analysis is based on it [25]. In chemical reaction terms, Eq (3) describes a first-order reaction kinetics in the infectious agent *I*. While Eq (3) points to a one-to-one transmission mechanism that is appealing in its simplicity, the reason that disease transmission may not be first-order regarding [*I*] is two-fold:

1. Due to contact structure heterogeneity, multiple transmission modes are more likely, with each having its own kinetics and reaction order. As in a chemical reaction with mixed kinetics [26], the overall, apparent reaction order would be a moving average of the mixed reaction orders, shifted by reaction conditions. During epidemics, movement restriction measures (e.g., travel restrictions) could change the kinetics of disease transmission, which is analogous to chemical reaction situations where an impediment of mass transfer changes the kinetics in diffusion-controlled chemical reactions and, subsequently, the overall reaction order [27].
2. As depicted in the SIR scheme, disease transmission is an autocatalytic process. The kinetic behavior of a wide range of autocatalytic systems can generally be expressed by the following scheme (see [28]):

A+nB→(1+n)B (4)

$$v=k[A]{\left[B\right]}^{n}$$ (5)

where *A* is the reactant, *B* is the product and catalyst, and *n* is the order of the autocatalytic reaction regarding *B*. Note that in this expression, *n* is not a stoichiometric coefficient as in a mass balance reaction equation. The chemical kinetics of autocatalytic chemical reactions has been extensively studied, with the reaction orders deduced from experimental data. Often, the reaction turned out to be non-first-order. For example, the decomposition of nitrobenzene derivatives manifested reaction orders (*n* values) ranging from 0.6 to 1.8 [29].

Available data on the COVID-19 pandemic suggest that there may be multiple transmission modes, including one to one or one to many [30,31]. Thus, the assumption contained in Eq (3) that the transmission is first-order with respect to [*I*] may not always hold and need to be made more general. Furthermore, mechanistically, a viral transmission does not always follow the molecularity [24] implied in Eq (3). To better reflect this, Eqs (1) and (2) can be modified as follows:

$$S+n\;I\overset{k}{\to }(n+1)I$$ (6)

$$I\overset{k_{r}}{\to }R$$ (7)

The rate law of the number of infectious cases after this modification becomes:

$$\frac{d[I]}{dt}=k[S]{\left[I\right]}^{n}-k_{r}[I],$$ (8)

where reaction order *n* is an empirical value to be extracted from observed data. Note that Eq (3) can be regarded as a special case of Eq (8), where disease transmission follows a first-order reaction kinetics with *n* = 1.

To further develop the modified mathematical model, it is necessary to establish a clear definition of [*I*]. In analogous chemical reaction terms, this is the concentration or density of infectious agents that are active in the population at a given time. The cumulative case density is not appropriate for this calculation, except at the beginning of an epidemic. During an epidemic, the infectious population is continuously filled by newly infected cases while simultaneously being drained by those in recovery or quarantine or who passed away. The effect of draining on [*I*] must be taken into consideration. The draining starts when infected individuals begin to recover or get quarantined after developing symptoms. However, in the early phase of an epidemic, almost no significant draining occurs, and consequently, *k*_*r*_ [*I*] is negligible. Therefore, we can eliminate the term, *k*_*r*_ [*I*] from Eq (8) and use cumulative confirmed case density for [*I*].

In addition, the infected population is extremely small relative to the total population in the early stage of an epidemic. Therefore, Eq (8) can be simplified further by approximating [*S*] as a constant, and the reaction as pseudo *n*^th^ order in [*I*]:

$$\frac{d[I]}{dt}=k[S]{\left[I\right]}^{n}=k'{\left[I\right]}^{n},$$ (9)

where *k’* = *k*[*S*].

Eq (9) can be mathematically solved. Integration gives the following equation:

$$\ln\left[I\right]\left\{ \begin{array}{c} \frac{\ln\left[(1-n\right]k^{\prime }]}{1-n}+\frac{1}{1-n}\ln t\;n\neq 1\\ {c+k}^{\prime }t\;n=1 \end{array}\right.$$ (10)

Note that Eq (10), when *n* is 1, becomes an exponential growth function. When *n* is not 1, then population-level epidemic data can be fitted as follows:

ln[I]=a+blnt, (11)

Where $a=\frac{\ln\left[(1-n\right)k^{\prime }]}{1-n}$ and $b=\frac{1}{1-n}$.

Thus, the modified SIR model shown in Eqs (10) and (11) expands the classic SIR model to include epidemic episodes with non-first-order transmission kinetics. Critical model coefficients, such as reaction order *k* and reaction order *n*, can be obtained by fitting this model to observed data. In the next section, we demonstrate this approach using the COVID-19 data.

## Data and methods

COVID-19 data are compiled by Our World in Data (ourworldindata.org) and available at https://github.com/owid/covid-19-data/tree/master/public/data. The data downloaded for the current study span from January 1, 2020 to June 30, 2020 and include the number of infected cases from 210 countries and independently administered regions. The terms, countries and regions, are interchangeably used for simplicity in the current study. The variables included in the data set are the number of confirmed cases, deaths, and tests conducted, as well as country-level variables concerning the demographic, economic, health, and disease control measures from each country.

All analyses were conducted using Python [32]. Random Forest (RF) regression analyses were conducted using Scikit-Learn (v0.22) developed for Python. Scikit-Learn is a free software machine learning library for the Python programming language (for more information, https://scikit-learn.org/stable/faq.html). Data and computing codes for the analyses reported in this article are available in an online repository [33].

## Results

### COVID-19 pandemic modeling

The relationship between *ln*[*I*] and *ln t* was examined using the COVID-19 data from 127 countries. Of the total 210 countries from the Our World Data, countries with a population size of at least one million and with at least one confirmed case per million were included (156 countries). Of those, we excluded data from 29 countries with fewer than three cases per million on the 14^th^ day since the first day of one confirmed case per million for concerns of data accuracy, thus resulting in *N* = 127 countries.

**Fig 3:**
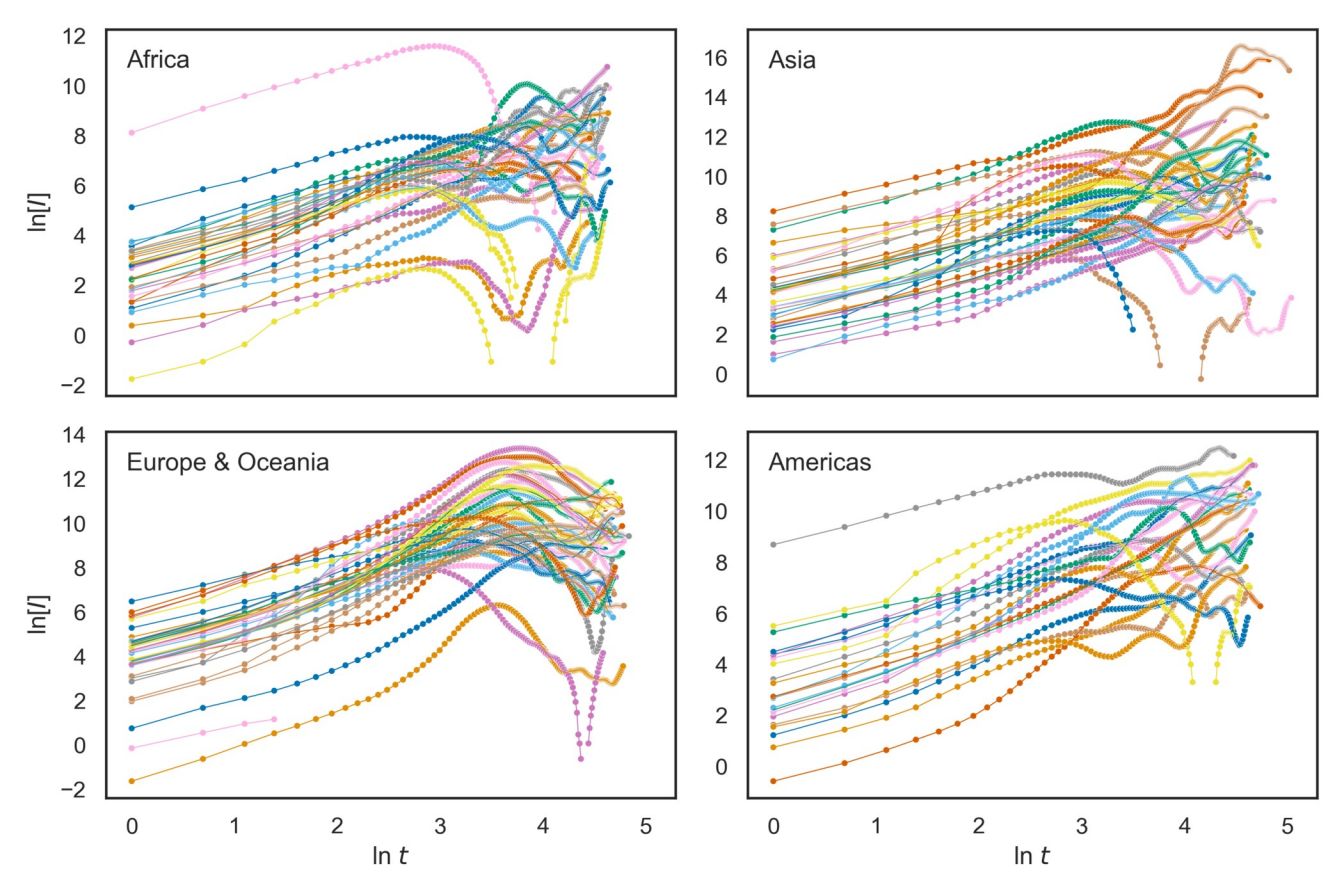
COVID-19 case development from the day that reached one confirmed case per million through June 30, 2020 (*N* = 127 countries). Y values correspond to the natural logarithm of the 14-day moving cumulative cases per million population per square kilometer, and X values stand for the natural logarithm of the number of days since one confirmed case per million (0 = the first day). Filled circles indicate observed values, and the lines connecting filled circles are interpolated lines between observed data points.

We defined the age of epidemic *t* a*s* the number of days since the day when there was at least one confirmed case per million population. In Fig 3, each line represents one country or region. Starting at around *t* = 14 days (at ~2.6 in *ln* scale), the curves of many countries showed downward bumps or inflection points. This timing coincides with the two-week isolation period recommended for people exposed to the SARS-Cov-2 virus. Conceivably, only after this time, the pool of infected individuals starts to be drained due to removal (i.e., recovery or quarantine). Therefore, data from the first 14 days were used in all subsequent analyses.

**Fig 4:**
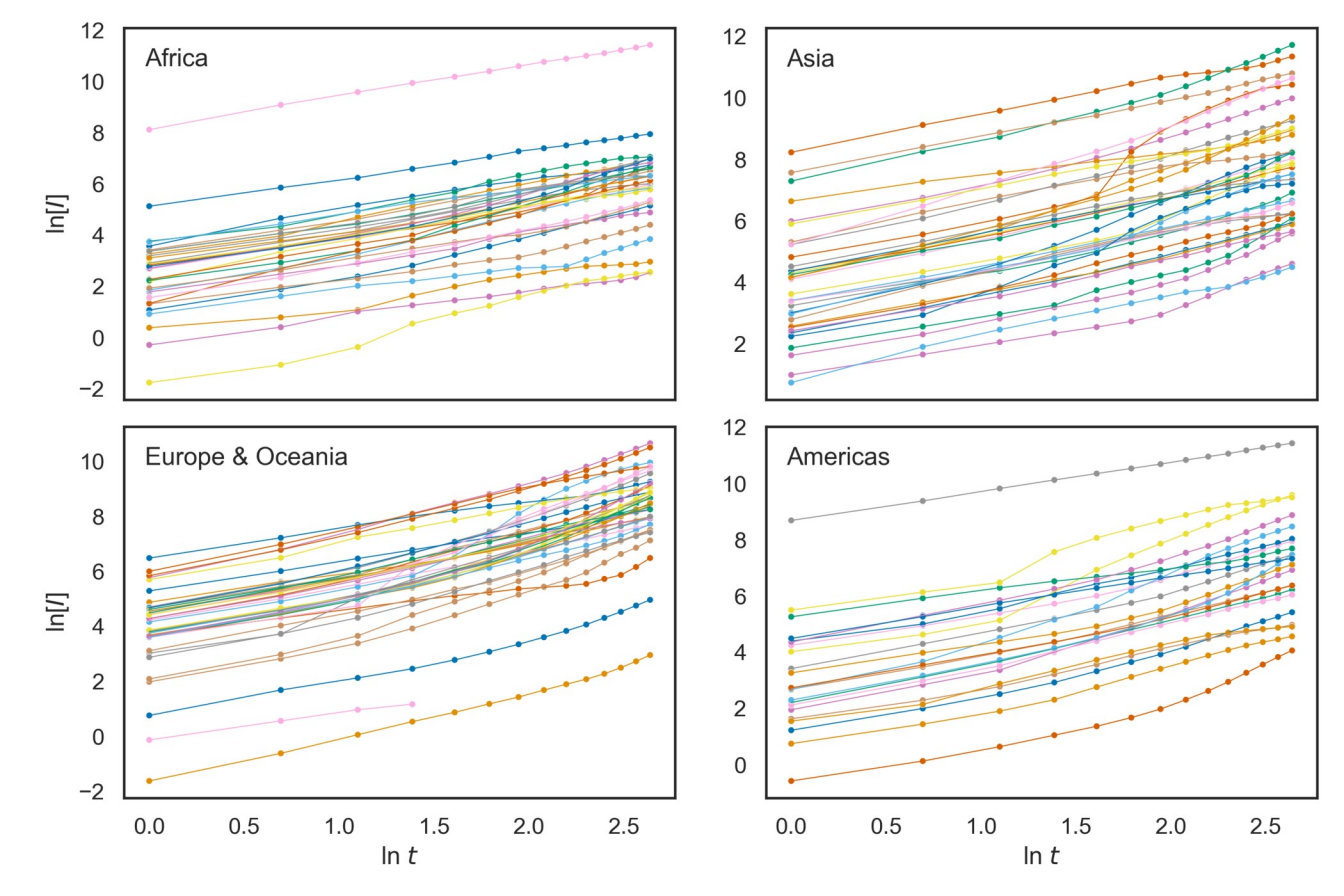
COVID-19 case development in each country during the first 14 days since reaching one confirmed case per million population (*N* = 127 countries). Y values correspond to the natural logarithm of the cumulative cases per million population per square kilometer, and X values stand for the natural logarithm of the number of days since reaching one confirmed case per million. Filled circles indicate observed values, and the lines connecting filled circles are interpolated lines between observed data points. S1 Fig in S1 File (see Supporting information) shows fitted regression lines and observed values.

The modified model in Eq (11) implies a linear relationship between ln[*I*] and ln *t* (i.e., a power growth function between [*I*] and *t*), rather than a linear relationship between ln[*I*] and *t* (i.e., an exponential function). Fig 4 suggests that for most countries, a linear regression between ln[*I*] and ln *t* would result in a nearly perfect fit.

**Fig 5:**
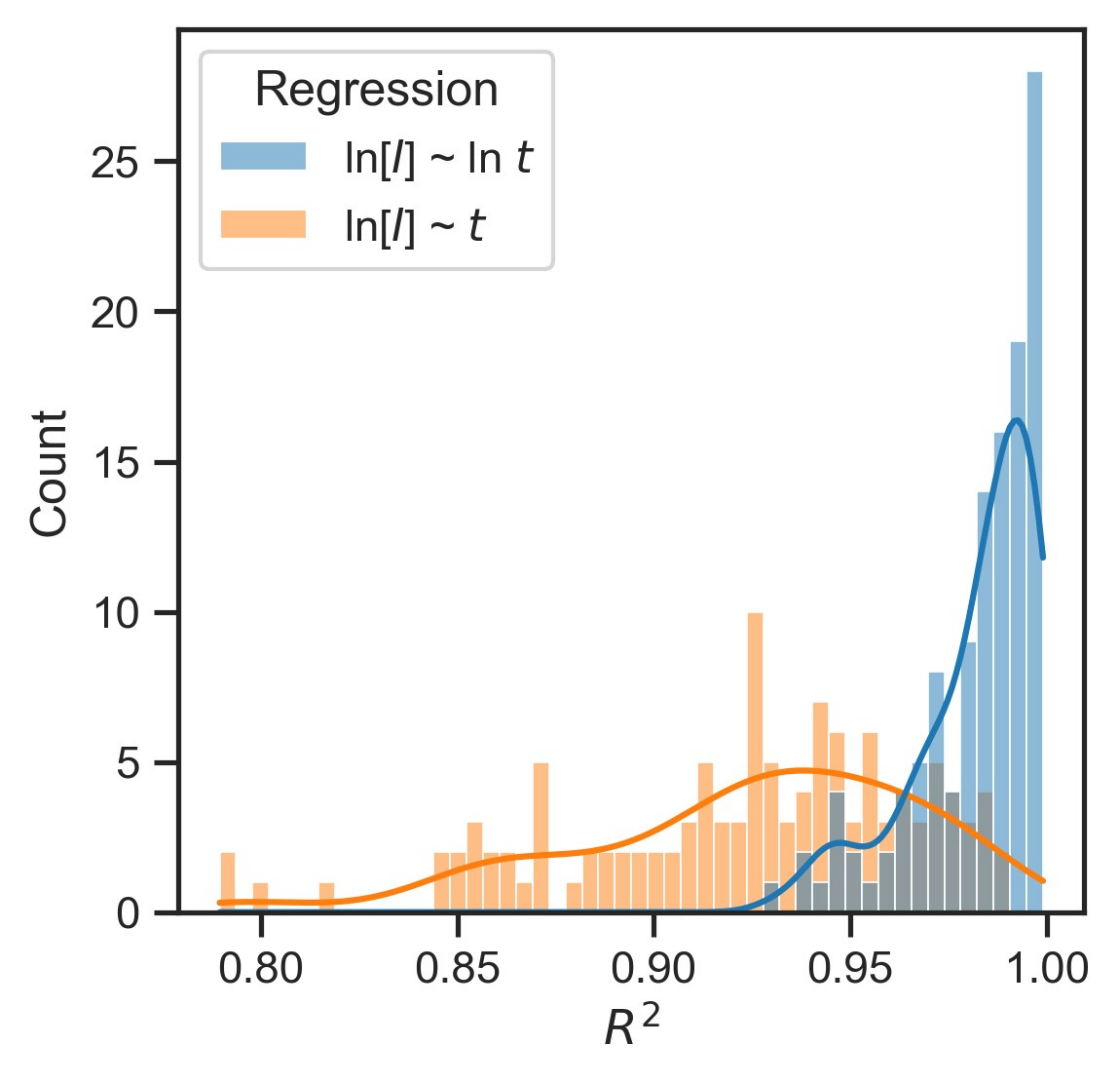
Histograms and kernel density estimation plots of *R*^*2*^ values from the modified mathematical model (in blue) and from the exponential model (in orange) for data from 127 countries. *R*^*2*^ values represent the extent to which a dependent variable is explained by the model. *R*^*2*^ = 1 is the upper bound value, indicating a perfect relationship. X-axis values represent the entire range of *R*^*2*^ values.

The modified model was fitted to data from each country. The resulting *R*^*2*^ values were mostly between 0.95 and 1 (Fig 5, blue). In contrast, the linear relationship between ln[*I*] and *t* entailed by the classic exponential model was comparatively less well-fitting for the same data, as shown by the distribution of *R*^*2*^ (Fig 5, orange). Both models estimated the same number of regression coefficients (i.e., intercept and regression slope). Therefore, the *R*^*2*^ distributions in Fig 5 provide comparable model fit information. The Akaike Information Criterion (AIC) and Bayesian Information Criterion (BIC) were -16.66 and -15.40, respectively, for the modified model; 8.63 and 9.89, respectively, for the classic exponential model.

Parameter estimates *a* and *b* for each country were obtained from the *ln*[*I*] ~ *ln t* regression: *a* and *b* are the intercept and slope, respectively, of the regression line. The *n* and *k’* values for each country were then calculated from *a* and *b* values using the equations previously defined (see Eq [11]). The transmission rate constant *k* for each country was further calculated by dividing *k’* by the population density [*S*] of that country (see Eq (9)).

A visual inspection of the distributions of *n* and *k* showed some potential outliers. We calculated the multivariate Mahalanobis distance metric to assess how far each country deviates from the center of the multivariate normal distribution, applied Chi-square tests for all distance metric values (for *df* = 1, *p* < 0.05), and consequently removed the following seven outliers: Estonia, Puerto Rico, Palestine, Jamaica, Belarus, Papua New Guinea, and Togo. Supporting information (see the S1 Appendix in S1 File) provides a detailed coverage on outliers, outlier detection methods [34], and outcomes.

**Fig 6:**
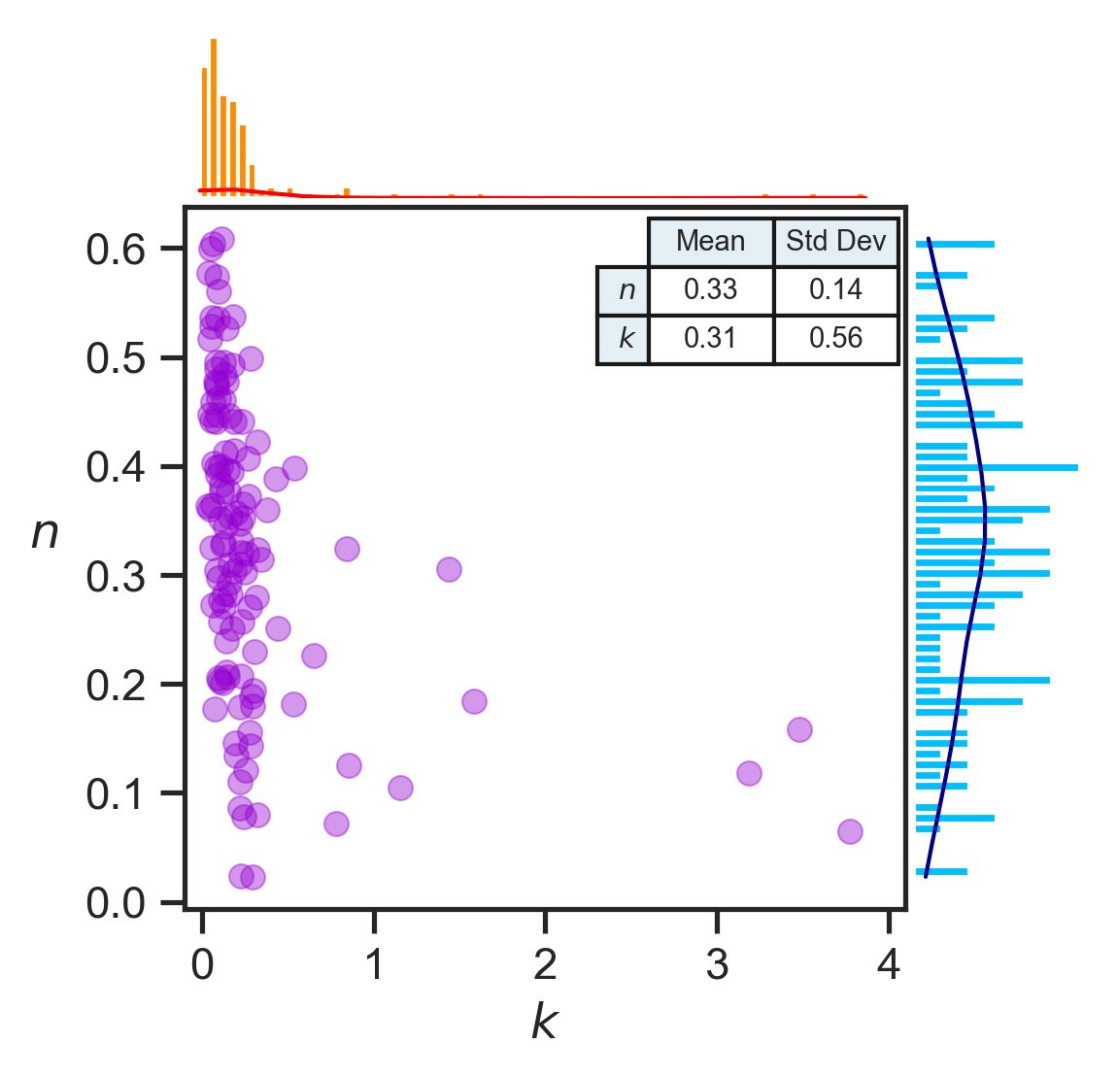
Statistics of the COVID-19 transmission rate constant *k* and reaction order *n* (*N* = 120 countries). The mean and standard deviation of *n* and *k* are shown in an inserted table in Fig 6. The bivariate distribution is shown in a scatter plot, and univariate distributions are shown on the top for *k* (in orange) and on the right for *n* (in blue).

Fig 6 shows the summary results after removing the seven countries identified by the multivariate Mahalanobis distance metric. The average transmission order *n* from 120 countries was 0.33, with a standard deviation of 0.14. The average transmission rate constant *k* was 0.31, with a standard deviation of 0.56.

For a chemical reaction, the reaction rate constant *k* is expressed by the following Arrhenius equation:

$$k=A\;e^{\frac{-E_{a}}{RT}},$$

where the pre-exponential factor *A* is a measure of how frequently collisions occur, and the exponential factor, e^−Ea/RT^, indicates the fraction of collisions with enough kinetic energy to lead to a reaction. Hence, the rate constant *k* gives the rate of successful collisions [24]. Intuitively, the counterpart parameter *k* in our model is also linked to the rate of "successful" disease transmission via interactions between infectious and susceptible individuals. Country-level differences in disease control measures, such as social distancing, as well as pre-COVID-19 conditions, would then result in noticeable country-to-country variations in *k*. The variance of *k* provides an opportunity to use statistical modeling or machine learning techniques to discover associations of transmission rates with country-level characteristics and country-specific disease-fighting measures.

We conducted a Random Forest (RF) analysis [35] using each country’s sociodemographic data and disease control measures as variables to predict epidemic-defining parameters *k* and *n*. RF uses a nonparametric, ensemble learning algorithm, which has been shown to yield significant improvements in prediction accuracy, compared with other algorithms, especially with a small sample with many features as we have here (see [36] for more explanations on this method). Significantly, RF can provide the relative Gini importance of each feature in predicting a dependent variable. In other words, RF can illustrate which country-level properties are relatively more closely associated with epidemic growth rates. Such information can be critical for formulating effective infection control strategies.

**Fig 7:**
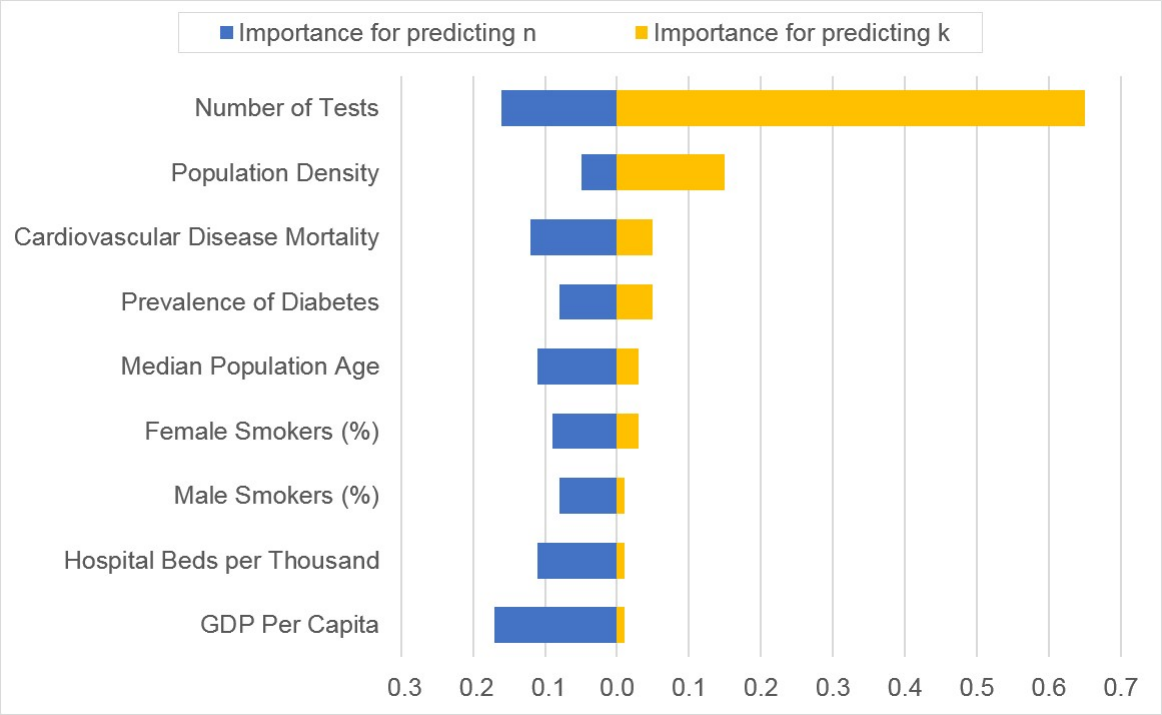
Importance of features in predicting *k* and *n* by Random Forest regression analyses. Importance values are scaled so that all blue bars add up to 1, and all orange bars add up to 1. There is no direct comparability between bars of different colors. Nine features with Importance greater than 0.01 were plotted in Fig 7. Population density: Number of people divided by land area, measured in square kilometers; Number of tests: Average daily tests for COVID-19 per 1,000 people; Median population age: Median age of the population, UN projection for 2020; Female smoker (%): Share of women who smoke, most recent year available; GDP per capita: Gross domestic product at purchasing power parity (constant 2011 international dollars); Cardiovascular disease mortality: Death rate from cardiovascular diseases in 2017 (annual number of deaths per 100,000 people); Prevalence of diabetes: Diabetes prevalence (% of population aged 20 to 79) in 2017; Hospital bed per thousand: Hospital beds per 1,000 people, most recent year available; Male smoker (%): Share of men who smoke, most recent year available.

Using RF models (25 trees with an automatic selection of the number of features), country-level features predicted *k* values well (training score *R*^*2*^ = 0.92; out-of-bag *R*^*2*^ = 0.49). Of all 14 features entered, we discovered that the number of COVID-19 tests conducted and population density had the most substantial impact on *k* (see Fig 7; horizontal orange bars). All other country-level features had less influence on *k*.

In contrast, country-specific data proved to be relatively weak predictors of *n* (175 trees, selecting a square root of the number of features), training score of *R*^*2*^ = 0.91 and out-of-bag *R*^*2*^ = 0.32). No feature stood out as critically important in predicting *n* (Fig 7; horizontal blue bars).

## Discussion

The prevailing pandemic modeling approaches contain the assumption of a first-order kinetics regarding [*I*], which mathematically leads to an exponential growth function. However, this assumption is unrealistic in many cases, as demonstrated here by real data. The current study developed a modified mathematical model (Eq (11)) derived from a different rate law (Eq (8)) with *n*^*th*^ reaction order, which is deduced from data. The results from the analysis of COVID-19 pandemic data suggest that this model provides a more accurate and inclusive description of the virus transmission dynamics.

The modified mathematical model provides two parameters describing an epidemic: transmission rate constant *k* and transmission reaction order *n*. When group-level heterogeneity exists, statistic or machine learning models can uncover hidden associations between group-specific features and outcome differences represented by *k*. For the COVID-19 pandemic data, the value *k* of each country appears to be strongly associated with control measures such as testing and environmental factors such as population density. Although Random Forest analysis results should not be interpreted as causal inference, such information provides valuable guidance for selecting effective disease control measures.

Transmission reaction order *n*, like its chemistry counterpart, appears to be more related to the reaction mechanism and less impacted by environmental factors. We observed that the *n* values of 120 different countries with very different characteristics were distributed narrowly around a mean of 0.33 with a standard deviation of 0.14 and a coefficient of variance of 0.42 (0.14/0.33). Meanwhile, country-level features were demonstrated to be relatively weak predictors of *n*. These findings suggest that *n* is a quantity pertaining to the intrinsic property of the epidemic agent.

Furthermore, Eq (6) can be transformed into the following equivalent form:

$$m\;S+I\overset{}{\to }(m+1)I,$$ (12)

where *m* = 1/*n*. The parameter *m* has its origin rooted in the “molecularity” of the disease transmission “reaction” through *n*. For the 120 countries in this study, *m* values ranged from 1.64 (Ireland) to 43.65 (Gabon), with a mean of 4.50. With coefficient *m*, Eq (12) better illustrates the process of disease transmission that occurs from one infectious to many susceptible individuals.

With traditional SIR models, data collected well into the epidemic development timeline are needed for establishing coefficients, β and γ [37,38]. In contrast, the modified model in this current study is uniquely suitable for extracting epidemic-defining parameters from sparse data at the onset of an epidemic outbreak. Insights afforded by the new mathematical model may be particularly valuable in guiding timely interventions at the most critical period of an epidemic.

More broadly, the non-first-order transmission kinetics model provides a general theoretical framework for epidemic modeling that complements the classic SIR model, where the rate law is implicitly assumed to be first order in the epidemic agent. In the current study, the country-level data used had a limited sample size. However, local-level data sharing similar environmental characteristics or data from different epidemics may provide a further test of the general applicability of the modified model. We also note that the range of *k* estimates was large, which may reflect important heterogeneity and can be explored further in future studies. Incomplete and noisy data might be other sources of the apparent heterogeneity, which makes it challenging to predict full epidemic trajectories [39]. Finally, the modified SIR model shown in the current study may be further fine-tuned and provide a building block for more elaborate epidemic models.

## Conclusion

The present paper provides a theoretical rationale for a modified mathematical epidemic model that removes an implicit assumption on reaction order in the classic SIR compartmental models to be more general, flexible, and accurate. More specifically, the modified mathematical model accommodates mixed-kinetics epidemics that are non-first-order and incorporates transmission heterogeneity. With this modified model, it is possible to derive critical epidemic-defining parameters early, which would be instrumental for understanding new epidemics and developing control measures.

## Supporting information

S1 File (DOCX)
