# Supplementary material for: An epidemic model for non-first-order transmission kinetics
Source: PLoS One. 2021 Mar 11;16(3):e0247512. doi: 10.1371/journal.pone.0247512 (PMC7951879; doi:10.1371/journal.pone.0247512)
Supplement: S1 File — (DOCX) [file pone.0247512.s001.docx]

**S1 Appendix: Outlier Detection Analysis**

**S1 Fig 1. COVID-19 case development in each country during the first 14 days since reaching one confirmed case per million population (*N* = 127 countries).** Y values correspond to the natural logarithm of the cumulative cases per million population per square kilometer, and X values stand for the natural logarithm of the number of days since reaching one confirmed case per million. Filled circles indicate observed values, and the solid lines are fitted regression lines for all countries. Figures were drawn with shared axes.


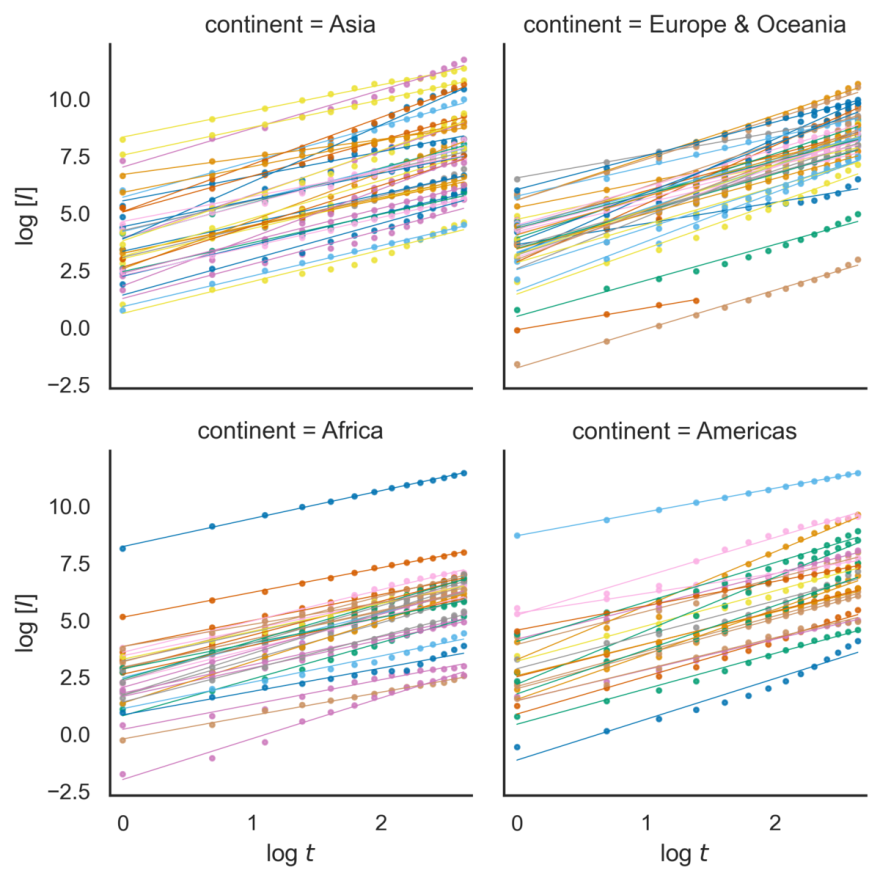


S1 Fig 2 shows the entire data points, including seven multivariate outliers (shown in gray filled circles).

**S1 Fig 2.** **Statistics of the COVID-19 transmission rate constant *k* and reaction order *n* (*N* = 127 countries).** The bivariate distribution is shown in a scatter plot, and univariate distributions are shown on the top for *k* (in orange) and on the right for *n* (in blue). Seven gray filled circles indicate the outliers identified and excluded in Figure 6.


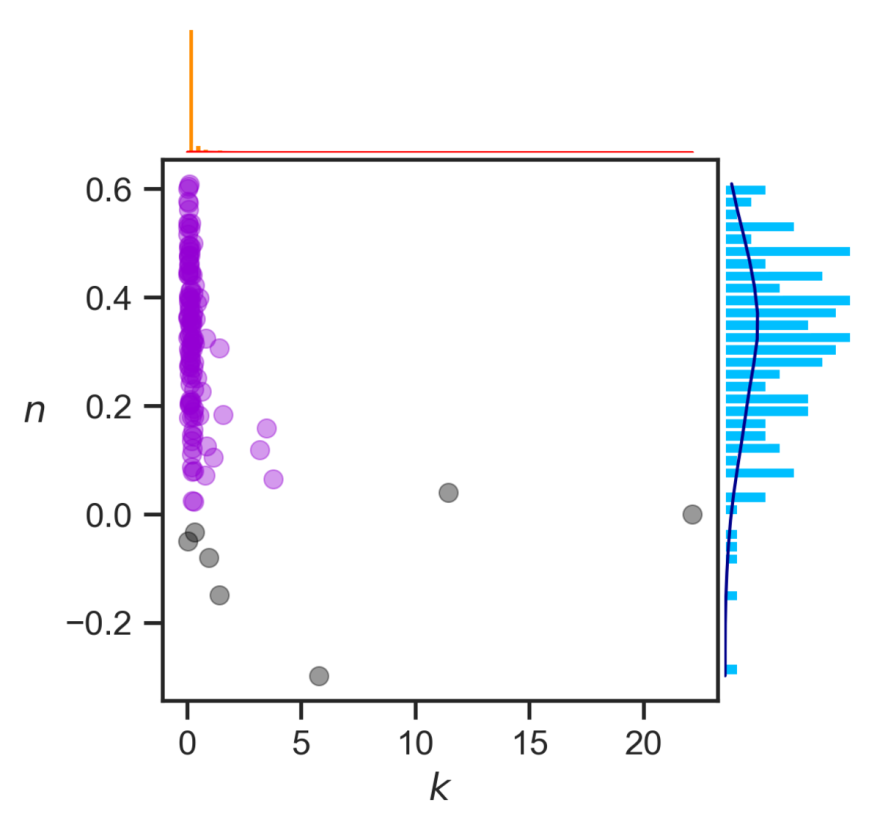


When plotting histograms for *n* and *k*, we first applied the outlier detection method proposed by Buzzi-Ferraris and Manenti (27) to the data, which starts removing the most extreme outlier until a predefined criterion is met. We found that this procedure was reasonable for detecting outliers for *n*, but not *k* estimates. The values for *k* were non-normally distributed with a heavy right tail (see S1 Fig 2). We deemed that the Buzzi-Ferraris and Manenti (2011) method is not ideal for the current data set because it is a univariate outlier detection procedure. In addition, the robust outlier detection approach (27) was developed with very large data sets in mind. It is well known that extreme values are more likely in small samples than large samples. Furthermore, in some countries, especially early in the pandemic, detecting and recording positive cases might not have been ideal. The wide range of *k* estimates may, hence, reflect data recording errors, extreme values in a small sample, and possible heterogeneous subgroups with different *k* values.

Hence, we calculated multivariate Mahalanobis distance (see S1 Fig 3) to detect multivariate outliers away from the center of the multivariate normal distribution, applied Chi-square tests, and consequently removed data from seven countries. The orange horizontal line indicates 3.84, a cutoff score for the Mahalanobis distance to be statistically significant at *p* < 0.05 with *df* = 1.
 **S1 Fig 3. Outlier identification based on the Mahalanobis distance metric.** Up to seven outliers (Estonia, Puerto Rico, Palestine, Jamaica, Belarus, Papua New Guinea, and Togo) were detected.


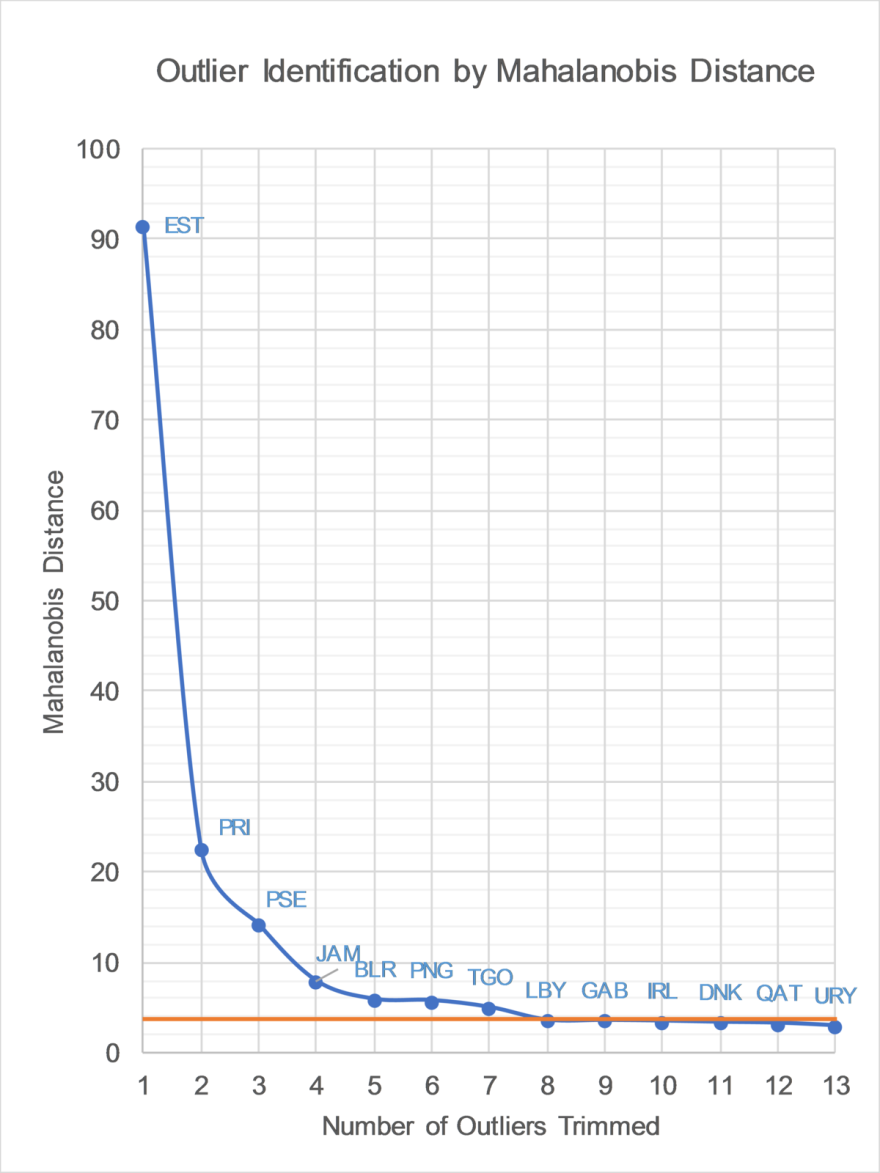


Based on the multivariate Mahalanobis distance metric, we removed seven countries, when plotting data for Figure 6. The omitted outliers were within the most extreme nine cases for *n* and within the most extreme 11 cases for *k*.

Note that when extreme values were removed successively using the Buzzi-Ferraris and Manenti method (27), resulting “clever means” and “clever standard deviations” after removing four extreme *n* and *k* values tended to approximate the reported mean and standard deviation of *n* and *k* (S1 Table 1), in part because six out of seven data points were detected by the two methods.

**S1 Table 1. After Removing Outliers from the Data Set**

|  | Mahalanobis Distance | |  | Buzzi-Ferraris and Manenti Method | |
| --- | --- | --- | --- | --- | --- |
|  | Mean | Std Dev |  | Mean | Std Dev |
| *n* | 0.33 | 0.14 |  | 0.32 | 0.15 |
| *k* | 0.31 | 0.56 |  | 0.29 | 0.48 |
